# Coarse-Grained Lattice Modeling and Monte Carlo Simulations of Stress Relaxation in Strain-Induced Crystallization of Rubbers

**DOI:** 10.3390/polym12061267

**Published:** 2020-06-01

**Authors:** Vladislav Egorov, Hiroshi Koibuchi, Chrystelle Bernard, Jean-Marc Chenal, Gildas Diguet, Gael Sebald, Jean-Yves Cavaille, Toshiyuki Takagi, Laurent Chazeau

**Affiliations:** 1 Cherepovets State University (ChSU), Prospekt Lunacharskogo, 5, 162600 Cherepovets, Vologda Oblast, Russia; rvladegorov@rambler.ru; 2 National Institute of Technology (KOSEN), Sendai College, 48 Nodayama, Medeshima-Shiote, Natori-shi, Miyagi 981-1239, Japan; 3 ELyTMaX UMI 3757, CNRS-Universite de Lyon, Tohoku University, International Joint Unit, Tohoku University, 2-1-1 Katahira, Aoba-ku, Sendai 980-8577, Japan; chrystelle.bernard@rift.mech.tohoku.ac.jp (C.B.); gildas.diguet.d4@tohoku.ac.jp (G.D.); gael.sebald@insa-lyon.fr (G.S.); jean-yves.cavaille@insa-lyon.fr (J.-Y.C.); 4 Frontier Research Institute for Interdisciplinary Sciences (FRIS), Tohoku University, 6-3 Aoba Aramaki, Aoba-ku, Sendai 980-8578, Japan; 5 Materials Engineering and Science (MATEIS), CNRS, INSA Lyon UMR 5510, Universite´ de Lyon Batiment B. Pascal, Avenue Jean Capelle, CEDEX, 69621 Villeurbanne, France; jean-marc.chenal@insa-lyon.fr (J.-M.C.); laurent.chazeau@insa-lyon.fr (L.C.); 6 Tohoku Forum for Creativity, Tohoku University, 2-1-1 Katahira, Aoba-ku Sendai 980-8577, Japan; toshiyuki.takagi.d4@tohoku.ac.jp

**Keywords:** rubber elasticity, strain-induced crystallization, stress relaxation, Monte Carlo, stress–strain curves, statistical mechanics

## Abstract

Two-dimensional triangulated surface models for membranes and their three-dimensional (3D) extensions are proposed and studied to understand the strain-induced crystallization (SIC) of rubbers. It is well known that SIC is an origin of stress relaxation, which appears as a plateau in the intermediate strain region of stress–strain curves. However, this SIC is very hard to implement in models because SIC is directly connected to a solid state, which is mechanically very different from the amorphous state. In this paper, we show that the crystalline state can be quite simply implemented in the Gaussian elastic bond model, which is a straightforward extension of the Gaussian chain model for polymers, by replacing bonds with rigid bodies or eliminating bonds. We find that the results of Monte Carlo simulations for stress–strain curves are in good agreement with the reported experimental data of large strains of up to 1200%. This approach allows us to intuitively understand the stress relaxation caused by SIC.

## 1. Introduction

**Figure 1:**
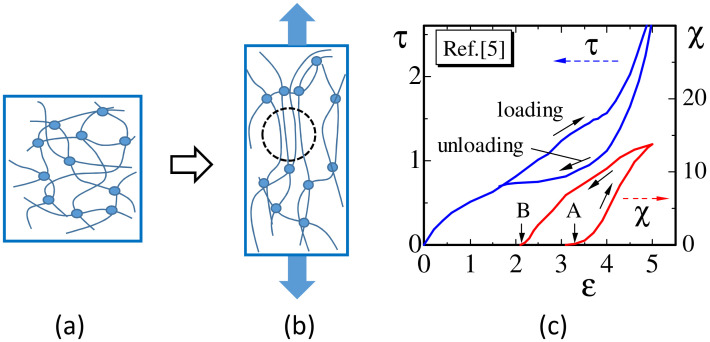
(**a**) The h state of rubber, where the polymer directions are isotropic, (**b**) extended or elongated state of rubber, where the polymer directions align along the tensile force direction and strain-induced crystallization (SIC) starts to appear, and (**c**) an illustration of stress–strain curves and crystallization ratio χ for loading and unloading processes influenced by SIC. Small circles in (**a**,**b**) represent cross-linkers. In (**c**), the letter A (B) denotes the point where crystallization starts (terminates) in the loading (unloading) process. The stress τ(MPa) and the crystallization ratio χ(%) in (**c**) are taken from experimental data in [5].

Natural rubbers undergo strain-induced crystallization (SIC), which attracts a lot of attention and has been studied extensively [1,2,3,4]. When the rubbers are stretched by an external tensile force, the entangled polymers partly change into an aligned state, and, then, this aligned state gradually converts into a crystalline state (Figure 1a,b). Moreover, if the stress is released after stretching or loading, one can also observe that the crystallized part immediately starts to melt. In this recovery or unloading process, hysteresis can be observed in the stress–strain curve and crystallization ratio (Figure 1c). This hysteresis indicates that the stress decreases due to the so-called crystallization-induced strain relaxation [3], which can be called stress relaxation. Such a stress relaxation is considered to be an equilibrium property because the stress–strain curve during the unloading process is close to the equilibrium curve. However, the stress relaxation is contradictory to our intuitive understanding that the mechanical strength is expected to be increased by the crystalline part of polymers, of which the mechanical strength is very large compared to the amorphous part, at least for intermediate strain region. Therefore, the stress relaxation by SIC should be studied in more detail to clarify how it can be understood by a simple modeling technique on the basis of statistical mechanics.

It is also interesting to see that the changes in state observed in the SIC process share the universal property with the first-order transition between gas and liquid; for example, [3]. From this first-order nature of SIC, we understand that the plateau behavior in the stress–strain curve for the unloading process has the same origin as that of the plateau in the pressure–density curve of the gas–liquid transition. On the other hand, the difference between SIC and the gas–liquid transition is that the crystallization is seen only partly in the case of rubbers, and this partial crystallization seems to occur due to the fact that the entangled polymers cannot be disentangled by the simple loading process from the topological constraint.

Therefore, in general, the effect of SIC on the stress–strain curves is very difficult to implement in the model [6]. In fact, it is easy to understand that the amorphous state is mechanically very different from the crystalline state [7,8,9,10,11,12]. Since the stress and strain of the crystalline state are expected to be completely different from those of the amorphous state, the mechanical response to external force is also completely different between the two states. For this reason, the tensile energy or Hamiltonian of the amorphous state cannot be shared by the crystalline state. This is one of the reasons why SIC is hard to study from the viewpoint of statistical mechanical modeling, where the Hamiltonian for each microscopic state should be defined explicitly.

In this paper, we use the tensile energy of the amorphous state of polymers simply by discarding the implementation of energy for the crystalline state. The tensile energy of the amorphous state is the so-called Gaussian bond potential, of which the one-dimensional version was originally assumed in the Gaussian chain model for polymers, where “bond” corresponds to the segment of the linear chain [13]. The two-dimensional (2D) version of the Gaussian bond potential is used for the triangulated surface models of membranes [14,15,16,17], and the 3D extension is straightforward with a tetrahedral lattice. In this paper, 2D and 3D versions of the Gaussian bond potential are assumed for our new SIC modeling. For these potential energies, the crystalline state can also be implemented simply by replacing elastic bonds with rigid bonds or empty bonds, where the rigid bond is a three-dimensional rigid body of fixed length and has no tensile energy, and the empty bond also has no tensile energy; these models are called the “rigid bond model” and “empty bond model”, respectively, in this paper. These two models are close to each other in the sense that no tensile energy is defined on the bond corresponding to the crystalline state.

It is well known from the thermodynamics of rubbers that rubber elasticity comes mainly from entropy [18,19]. Since this entropy contribution is a major part of the elasticity, the rubber elasticity is called entropy elasticity. However, we study rubber elasticity by statistical mechanical models in which two different microscopic variables are assumed, and we will not go into the details of thermodynamic theory.

## 2. Models

### 2.1. 2D Lattice

**Figure 2:**
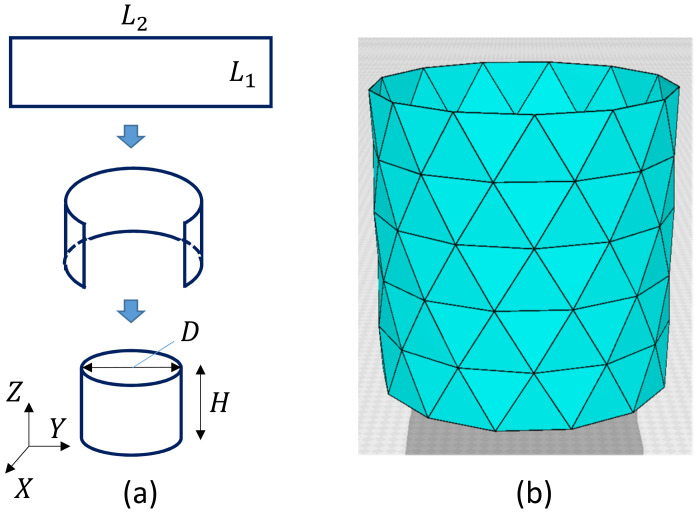
(**a**) A cylindrical surface is made of a rectangular surface of size $L_{1}\;\times \;L_{2}$, where $L_{1}$ and $L_{2}$ are integers, and (**b**) a triangulated cylinder of size N=78, where the total number of boundary vertices is $N_{\mathrm{bnd}}\;=2\left(L_{2}\;-\;1\right)\;=\;26$. Small numbers $L_{1}\left(=\;6\right)$ and $L_{2}\left(=\;14\right)$ are assumed here to visualize triangles clearly. The height $H_{0}$ and the diameter *D* are given by Equation (1) with the edge length *a*.

A 2D triangulated lattice was originally constructed for the surface model of membranes [14,15,16,17]. Here, we use cylindrical lattices for the calculation of surface tension by fixing the boundary of the cylinder, which will be shown in the following subsection. The lattice size *N*, which is the total number of vertices including the boundary vertices, is given by $N\;=\;L_{1}\left(L_{2}\;-\;1\right)$, where $L_{1}$ and $L_{2}$ are the total number of vertices on the edges of the rectangular plate (Figure 2a). The height $H_{0}$ and the diameter *D* of the cylinder are assumed to be the same such that

(1) $$H_{0}=D=\left(\sqrt{3}/2\right)\left(L_{1}-1\right)a,$$

where *a* is the lattice spacing or can be called the edge (or bond) length of the initial lattice. The lattice spacing is fixed to a=1 and eliminated from the expressions of bond length henceforth; however, this spacing is restored if the units of surface tension are changed from the simulation units to the physical units in a later section. The diameter *D* is given by $\pi D\;=\;(L_{2}\;-\;1)a$. Therefore, from $D\;=\;H_{0}$, the relation $L_{2}\;=\;\left(\sqrt{3}\pi /2\right)\left(L_{1}\;-\;1\right)\;+\;1$ is the condition for the triangles to be the regular triangle. A snapshot of cylinders of size $L_{2}\;=\;14$, $L_{1}\;=\;6$, and N=78 is shown in Figure 2b, where the condition $L_{2}\;=\;\left(\sqrt{3}\pi /2\right)\left(L_{1}\;-\;1\right)\;+\;1$ is not always exactly satisfied because the right-hand side is not an integer.

### 2.2. Rigid Bond Model and Empty Bond Model

**Figure 3:**
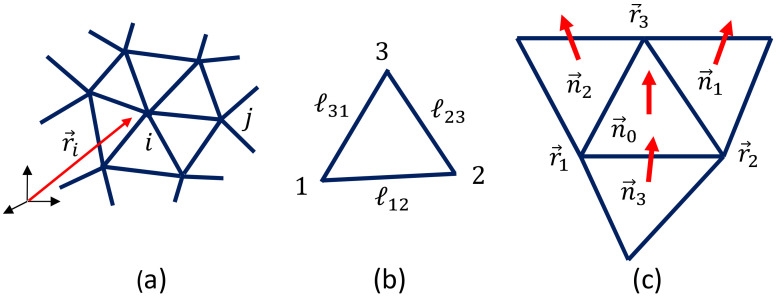
(**a**) A triangular mesh or lattice and the vertex position ${\vec{r}}_{i}$, (**b**) a triangle with vertices 1,2,3 and edge lengths $\ell_{12}$, $\ell_{23}$, $\ell_{31}$, and (**c**) the unit normal vectors ${\vec{n}}_{0}$ of the triangle 123, and those ${\vec{n}}_{i},\left(i\;=\;1,2,3\right)$ of its neighboring triangles, where the suffix *i* denotes the corresponding triangle.

Let ${\vec{r}}_{i}\left(\in R^{3}\right),\left(i\;=\;1,\cdots ,N\right)$ be the vertex position of the lattice (Figure 3a), $\ell_{ij}\;=\;\left|{\vec{r}}_{i}\;-\;{\vec{r}}_{j}\right|$ be the bond length (Figure 3b), and ${\vec{n}}_{i}$ be the unit normal vector of the triangle (Figure 3c). These variables ${\vec{r}}_{i}$ are integrated into the partition function *Z* for 2D models (here, we discuss 2D surface models), and *Z* is given by

(2) $$Z\left(\ell_{0}\right)=\sum_{\sigma }\int \prod_{i=1}^{N-N_{\mathrm{bnd}}}d{\vec{r}}_{i}\prod_{i=1}^{N_{\mathrm{bnd}}}d{\vec{r}}_{i}\exp\left(-S\left(\vec{r},\sigma \right)\right),$$

where the symbol $\ell_{0}$ in $Z(\ell_{0})$ indicates that the model implicitly depends on a constant $\ell_{0}$, which will be used to define the crystalline bond below. The symbol $\int \prod_{i=1}^{N-N_{\mathrm{bnd}}}d{\vec{r}}_{i}$ denotes the 3D integration for the internal vertices. The other $\int \prod_{i=1}^{N_{\mathrm{bnd}}}d{\vec{r}}_{i}$ denotes 2D integration for the boundary vertices on the upper and lower boundaries with a periodic boundary condition such that

(3) $$Z_{i}^{U}=Z_{i}^{L}+H,\;X_{i}^{2}+Y_{i}^{2}=D^{2}/4,$$

where $Z_{i}^{U}$ and $Z_{i}^{L}$ denote the *Z* component of the vertex position ${\vec{r}}_{i}=\left(X_{i},Y_{i},Z_{i}\right)$ on the upper and lower boundaries (no confusion is expected for the symbol of the partition function and that of the coordinate symbol). The second equation in Equation (3) denotes a constraint that the diameter of the boundary circle is given by *D* (Figure 2a). The reason why $\int \prod_{i=1}^{N_{\mathrm{bnd}}}d{\vec{r}}_{i}$ becomes a 2D integration is that the boundary vertices are allowed to move not only along the boundary circle of fixed diameter *D*, but also along the *Z* direction due to the periodic boundary condition. For the 3D thick cylinder model, $\int \prod_{i=1}^{N_{\mathrm{bnd}}}d{\vec{r}}_{i}$ becomes a one-dimensional integration because no periodic boundary condition is imposed.

The symbol σ in Equation (2), which has values in {0,1}, is another dynamical variable defined on the bond *i*. The value of σ represents whether the bond *i* is rigid or amorphous:

(4) $$\begin{array}{c} \sigma_{i}=\left\{\begin{array}{cc} 0 & \left(\Leftrightarrow \mathrm{bond}\;i\;=\;\mathrm{amorphous}\right)\\ 1 & \left(\Leftrightarrow \mathrm{bond}\;i\;=\;\mathrm{rigid}\right) \end{array}\right.\;\;,\;\left(\mathrm{rigid}\;\mathrm{bond}\;\mathrm{model}\right). \end{array}$$

The rigid bond is a rigid body, which is allowed to move by 3D rotation and translation. In other words, two vertices *i* and *j*, which are connected by a rigid bond, move with a constraint $\ell_{ij}\left(=\;\right|{\vec{r}}_{i}\;-\;{\vec{r}}_{j}\left|\right)\;=\;\ell_{ij}^{c}$, where $\ell_{ij}^{c}$ is described in the following paragraph. In contrast, this constraint on $\ell_{ij}$ is not imposed on any two vertices connected by an amorphous bond. Another model is the “empty bond” model, which is defined by replacing “rigid” with “empty” in Equation (4) such that

(5) $$\begin{array}{c} \sigma_{i}=\left\{\begin{array}{cc} 0 & \left(\Leftrightarrow \mathrm{bond}\;i\;=\;\mathrm{amorphous}\right)\\ 1 & \left(\Leftrightarrow \mathrm{bond}\;i\;=\;\mathrm{empty}\right) \end{array}\right.\;\;,\;\left(\mathrm{empty}\;\mathrm{bond}\;\mathrm{model}\right). \end{array}$$

**Figure 4:**
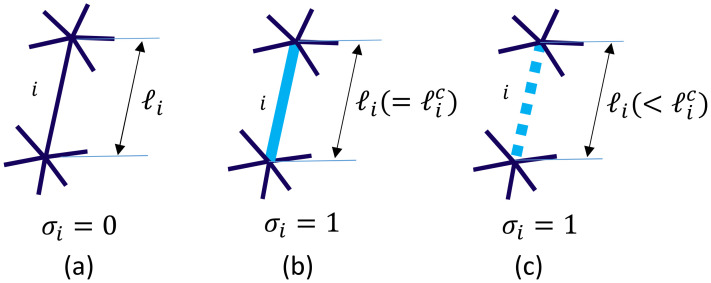
Illustration of the bond *i* for (**a**) $\sigma_{i}\;=\;0$ corresponding to the amorphous bond, (**b**) $\sigma_{i}\;=\;1$ corresponding to the rigid bond, and (**c**) $\sigma_{i}\;=\;1$ corresponding to the empty bond.

The variable $\sigma_{i}$ in Equations (4) and (5) can be changed from $\sigma_{i}\;=\;0$ to $\sigma_{i}\;=\;1$ when the bond length $\ell_{i}$ is $\ell_{i}\;>\;\ell_{0}$ using the constant $\ell_{0}$ in $Z(\ell_{0})$ according to the Monte Carlo update procedure described in a later section. If $\sigma_{i}$ is changed from $\sigma_{i}\;=\;0$ to $\sigma_{i}\;=\;1$, then the bond length $\ell_{i}$ is fixed to the length $\ell_{i}^{c}$, which is the length of bond *i* just before the change in $\sigma_{i}$ in the rigid model. In the case of the empty bond model, the length $\ell_{i}$ of the crystalline bond is changeable to a new length $\ell_{i}^{\prime }$ if $\ell_{i}^{\prime }<\ell_{i}^{c}$, and this $\ell_{i}^{c}$ is also changeable depending on the update of $\sigma_{i}$, which is the same as in the case of the rigid bond model. The constraint $\ell_{i}^{c}>\ell_{0}$ is always satisfied in both models. The empty bond is almost close to the rigid bond except that the length $\ell_{i}$ is changeable to $\ell_{i}^{\prime }<\ell_{i}^{c}$. This fact implies that the empty bond length $\ell_{i}$ is also prohibited from extending to $\ell_{i}^{\prime }\;>\;\ell_{i}^{c}$. The bond configurations for amorphous, rigid, and empty bonds are illustrated in Figure 4a–c.

We should comment that the total number of degrees of freedom of variable $\vec{r}$ is slightly reduced by crystallization. Indeed, in the rigid bond model, a crystalline bond is allowed to move by three-dimensional translation and two-dimensional rotation, where the rotational degrees of freedom are given as 2 because the bond is a one-dimensional object. Therefore, the 6 degrees of freedom, which is the sum of the degrees of freedom for translations of the terminal vertices of the bond, is reduced to 5 by crystallization. Thus, the total number of degrees of freedom is reduced by $N_{\mathrm{cr}}$, which is the total number of crystalline bonds, in the rigid bond model. In the case of the empty bond model, the total number of reductions in the degrees of freedom of variable $\vec{r}$ is effectively considered to be $N_{\mathrm{cr}}/2$ because the crystalline bond length is allowed to shrink even though the extension is not allowed, and, for this reason, the reduction per crystalline bond is roughly estimated to be 1/2.

The Hamiltonian for the rigid and empty bond models is given by

(6) $$\begin{array}{c} \begin{array}{c} S=S_{1}+bS_{2}+\kappa S_{3}+U_{1}+U_{2},\\ S_{1}=\frac{1}{2}\sum_{\Delta }S_{1\Delta },\;S_{1\Delta }=\ell_{12}^{2}+\ell_{23}^{2}+\ell_{31}^{2},\\ S_{2}=\frac{1}{2}\sum_{\Delta }\left(S_{2\Delta }^{\left(1\right)}+S_{2\Delta }^{\left(2\right)}\right),\;S_{2\Delta }^{\left(1\right)}=\ell_{12}^{4}+\ell_{23}^{4}+\ell_{31}^{4},\;S_{2\Delta }^{\left(2\right)}=2\left(\ell_{12}^{2}\ell_{13}^{2}+\ell_{21}^{2}\ell_{23}^{2}+\ell_{32}^{2}\ell_{31}^{2}\right),\\ \ell_{ij}=\left\{\begin{array}{cc} |{\vec{r}}_{j}-{\vec{r}}_{i}| & \left(\sigma =0\right)\\ 0 & \left(\sigma =1\right) \end{array}\right.\;\;,\\ S_{3}=\frac{1}{2}\sum_{\Delta }\left(S_{3\Delta ,1}+S_{3\Delta ,2}+S_{3\Delta ,3}\right),\;S_{3\Delta ,i}=\left\{\begin{array}{cc} 1-{\vec{n}}_{0}\cdot {\vec{n}}_{i} & \left(\sigma =0\right)\\ 1-{\vec{n}}_{0}\cdot {\vec{n}}_{i}\;\left(\mathrm{rigid}\right)\;\mathrm{or}\;0\;\left(\mathrm{empty}\right)\; & \left(\sigma =1\right) \end{array}\right.\;\;, \end{array} \end{array}$$

and

(7) $$\begin{array}{c} \begin{array}{c} U_{1}=\sum_{\mathrm{bond}\;i}U_{1i}\left(\vec{r},\sigma \right),\;U_{1i}\left(\vec{r},\sigma \right)=\left\{\begin{array}{cc} \infty & \left(\sigma_{i}=1\;\&\;\ell_{i}\neq \ell_{i}^{c}:\mathrm{rigid}\right)\;\mathrm{or}\;\left(\sigma_{i}=1\;\&\;\ell_{i}>\ell_{i}^{c}:\mathrm{empty}\right)\\ 0 & \left(\mathrm{otherwise}\right) \end{array}\right.\;\;,\\ U_{2}=\sum_{\mathrm{bond}\;i}U_{2i}\left(\vec{r},\sigma \right),\;U_{2i}\left(\vec{r},\sigma \right)=\left\{\begin{array}{cc} \infty & \left(\sigma_{i}=\sigma_{j}=1\;\&\;i\;\mathrm{and}\;j\;\mathrm{are}\;\mathrm{connected}\right)\\ 0 & \left(\mathrm{otherwise}\right) \end{array}\right.\;\;, \end{array} \end{array}$$

where the first two terms $S_{1}\;+\;bS_{2}$ and the third term $\kappa S_{3}$ correspond to the tensile energy and the bending energy, respectively, and the final two terms $U_{1}\;+\;U_{2}$ are constraint potentials. The symbol σ in the definition of $\ell_{ij}$ is the variable σ on the bond connecting the vertices *i* and *j*. No difference between the rigid and empty bond models is in the definition of $\ell_{ij}$ except that the length $\ell_{ij}$ of crystalline bond ij is allowed to be $\ell_{ij}\;<\;\ell_{ij}^{c}$ in the case of the empty bond model. In contrast, the length of bond ij is prohibited from being $\ell_{ij}\;>\;\ell_{ij}^{c}$ for $\sigma_{ij}\;=\;1$ in both models. In this sense, this definition of $\ell_{ij}$ is specific to the models for SIC in this paper. In the bending energy $S_{3\Delta ,i}\left(i\;=\;1,2,3\right)$, σ is defined on the bond shared by triangles 0 and *i* (see Figure 3c). This definition of the bending energy is also specific to the models for SIC in this paper. These specific definitions of $\ell_{ij}$ and $1\;-\;{\vec{n}}_{i}\cdot {\vec{n}}_{j}$ come from the fact that the crystalline state is implemented in the context of the Gaussian bond model for polymers [13].

Due to the factor 1/2, the Gaussian bond potential $S_{1}$ is identical to $S_{1}\;=\;\sum_{\mathrm{bond}\;i}\ell_{i}^{2}$, which is given by the sum of bond length squares $\ell_{i}^{2}$. The reason why the sum $\sum_{\Delta }$ is used for $S_{1}$ instead of $\sum_{\mathrm{bond}\;i}$ is because the discrete form of the quadratic term $S_{2}$ is naturally given by $\sum_{\Delta }$, and the same summation convention is used for $S_{1}$. The numerical factor 1/2 in $S_{2}$ is determined such that it is identical to the factor in $S_{1}$.

**Figure 5:**
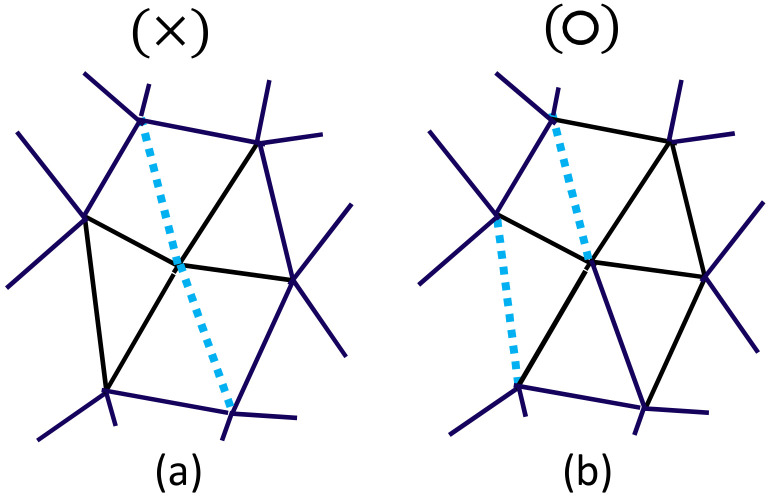
(**a**) A configuration that is not allowed because the two bonds of σ=1 are connected, and (**b**) a configuration that is allowed because the two bonds of σ=1 are separated. The maximal value $\chi_{\max}$ of the crystallization ratio of Equation (8) is not given as an input parameter in the simulations, but it is automatically determined by the constraint illustrated in the figure.

Note that $U_{1}$ is the constraint potential for $\ell_{i}$ in both models. From this constraint $U_{1}$, the length $\ell_{i}$ of the empty bond is only allowed to be $\ell_{i}\;\leq \;\ell_{i}^{c}$, as mentioned above; however, this is strictly prohibited for the rigid bond because $\ell_{i}$ should always satisfy $\ell_{i}\;=\;\ell_{i}^{c}$ from the definition of $\ell_{i}$. The second constraint $U_{2}$ prohibits the crystalline bond *i* from being consecutively connected (Figure 5a), or, in other words, every crystalline bond *i* must be isolated or linked to the amorphous bond *j* (Figure 5b). This constraint $U_{2}$ also plays a role in the constraint on the total number of crystalline bonds because the maximum number of crystalline bonds is limited by a lattice geometric (or topological) reason.

As a consequence of the constraint $U_{2}$, no constraint is imposed on the upper limit for the crystallization ratio χ such that $\chi \;\leq \;\chi_{\max}$, where χ is defined by

(8) $$\chi =\left(1/N_{B}\right)\sum_{\mathrm{bond}\;i}\sigma_{i},$$

where $\sum_{i}\sigma_{i}$ is the total number of crystalline bonds and $N_{B}\left(=\;\sum_{i}1\right)$ is the total number of bonds. The value of $\chi_{\max}$ depends on the dimension of the lattice, that is, whether it is a 2D or 3D lattice; however, in both cases, $\chi_{\max}$ is reasonable in the sense that it is close to the experimental value, such as $\chi_{\max}\left(\mathrm{Exp}\right)\;≃0.1\;$ [1,2,3,4], which will be shown in the presentation section.

The mean value 〈Q〉 of physical quantity $Q(\vec{r},\sigma )$ (where the symbol *Q* denotes an arbitrary quantity) is given by

(9) $$\begin{array}{c} \langle Q\rangle =\sum_{\sigma }\int \prod_{i=1}^{N-N_{\mathrm{bnd}}}d{\vec{r}}_{i}\prod_{i=1}^{N_{\mathrm{bnd}}}d{\vec{r}}_{i}Q\left(\vec{r},\sigma \right)\exp\left(-S\left(\vec{r},\sigma \right)\right)/Z. \end{array}$$

We simply write the mean value *Q* by removing the symbol 〈·〉 henceforth. To calculate these multiple integrations and the sum $\sum_{\sigma }$ over all possible configurations of σ, we use Metropolis Monte Carlo (MC) simulations, which are described in the following section. Note that all physical quantities are calculated with the dynamical variables $\vec{r}$ and σ, which are the vertex position $\vec{r}\;=\;\left\{{\vec{r}}_{1},\ldots ,{\vec{r}}_{N}\right\}$ and the spin variable at the vertices $\sigma \;=\;\{\sigma_{1},\ldots ,\sigma_{N}\}$, respectively.

The 2D continuous forms of the Hamiltonian corresponding to the discrete $S_{1}$ and $S_{2}$ in Equation (6) are given by

(10) $$\begin{array}{c} S_{1}=\int \sqrt{g}d^{2}xg^{ab}\frac{\partial \vec{r}}{\partial x^{a}}\cdot \frac{\partial \vec{r}}{\partial x^{b}},\;S_{2}=\int \sqrt{g}d^{2}x{\left(g^{ab}\frac{\partial \vec{r}}{\partial x^{a}}\cdot \frac{\partial \vec{r}}{\partial x^{b}}\right)}^{2}, \end{array}$$

where $g^{ab}$ is the inverse of a metric tensor $g_{ab}$ given by the 2×2 matrix, and *g* is the determinant. To obtain the discrete Hamiltonian, we simply assume that $g_{ab}\;=\;\delta_{ab}$, which is called the Euclidean metric. By the replacements $\partial_{1}\vec{r}\left(=\partial \vec{r}/\partial x^{1}\right)\to {\vec{r}}_{2}-{\vec{r}}_{1}$ and $\partial_{2}\vec{r}\to {\vec{r}}_{3}-{\vec{r}}_{1}$ (see Figure 3b), and by the symmetrization of the obtained expression of the Hamiltonian due to the three possible local coordinate origins in a triangle, we have the discrete $S_{1}$ and $S_{2}$ in Equation (6). More detailed information on the discretization is given in Ref. [20].

### 2.3. 3D Model

**Figure 6:**
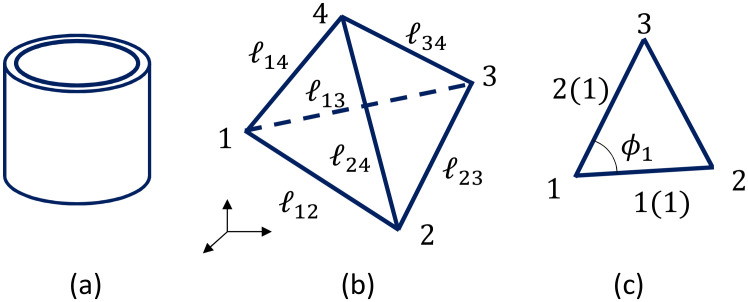
(**a**) A cylinder for the 3D model, (**b**) a tetrahedron with vertices 1,2,3,4 and the corresponding edge length $\ell_{ij},\left(i\;\neq \;j\;=\;1,\;\ldots \;,4\right)$, and (**c**) an internal angle $\phi_{1}$ of the triangle 123, where 1(1) and 2(1) are the edges, of which the internal angle is $\phi_{1}$.

The three-dimensional (3D) continuous Hamiltonian is obtained from the 2D version in Equation (10) simply by replacing the 2D integration $\int \sqrt{g}d^{2}x$ with the 3D integration $\int \sqrt{g}d^{3}x$. In the 3D case, the metric tensor $g_{ab}$ is also replaced by a 3×3 matrix. Thus, these 3D continuous $S_{1}$ and $S_{2}$ can also be converted to discrete forms on the 3D lattice, which is a thick cylinder (Figure 6a) discretized by tetrahedrons (Figure 6b). On this thick cylinder, the vertices of tetrahedrons are distributed only on the inner and outer surfaces, and, therefore, the thickness is negligible compared with the diameter. Note also that in the 3D case, only the empty bond model is studied. The reason is that, as we will see in the presentation section, there is almost no difference between the results of the 2D empty and rigid models, or the 2D empty model is slightly better for the stress relaxation behavior, and, moreover, the empty model is more simple than the rigid model in its definition.

The 3D version of the constraint potentials $U_{1}$ and $U_{2}$ has the same expressions as those in Equation (7), and, hence, their expressions are not written below.

(11) $$\begin{array}{c} \begin{array}{c} S=S_{1}+bS_{2}+\kappa S_{3}+U_{1}+U_{2},\\ S_{1}=\frac{1}{\bar{N}}\sum_{\mathrm{tet}}S_{1\;\mathrm{tet}},\;S_{1\;\mathrm{tet}}=\ell_{12}^{2}+\ell_{13}^{2}+\ell_{14}^{2}+\ell_{23}^{2}+\ell_{24}^{2}+\ell_{34}^{2},\\ S_{2}=\frac{1}{\bar{N}}\sum_{\mathrm{tet}}\left(S_{2\;\mathrm{tet}}^{\left(1\right)}+S_{2\;\mathrm{tet}}^{\left(2\right)}\right),\;S_{2\;\mathrm{tet}}^{\left(1\right)}=\ell_{12}^{4}+\ell_{13}^{4}+\ell_{14}^{4}+\ell_{23}^{4}+\ell_{24}^{4}+\ell_{34}^{4},\\ S_{2\;\mathrm{tet}}^{\left(2\right)}=2\left(\ell_{12}^{2}\ell_{13}^{2}+\ell_{12}^{2}\ell_{14}^{2}+\ell_{13}^{2}\ell_{14}^{2}+\ell_{21}^{2}\ell_{23}^{2}+\ell_{21}^{2}\ell_{24}^{2}+\ell_{23}^{2}\ell_{24}^{2}\right.\\ \;\;\left.+\ell_{31}^{2}\ell_{32}^{2}+\ell_{31}^{2}\ell_{34}^{2}+\ell_{32}^{2}\ell_{34}^{2}+\ell_{41}^{2}\ell_{42}^{2}+\ell_{41}^{2}\ell_{43}^{2}+\ell_{42}^{2}\ell_{43}^{2}\right),\\ \ell_{ij}=\left\{\begin{array}{cc} |{\vec{r}}_{j}-{\vec{r}}_{i}| & \left(\sigma =0\right)\\ 0 & \left(\sigma =1\right) \end{array}\right.\;\;,\\ S_{3}=\sum_{\mathrm{triangle}\;i}S_{3,i},\\ S_{3,i}=\left\{\begin{array}{cc} 1-\cos(\phi_{i}-\pi /3) & \left(\sigma_{1(i)}\;=\;0\;\mathrm{and}\;\sigma_{2(i)}\;=\;0,\;\mathrm{or}\;\mathrm{empty}\right)\\ 0 & \left(\mathrm{otherwise}\right) \end{array}\right.\;\;, \end{array} \end{array}$$

where $\sum_{\mathrm{tet}}$ in $S_{1}$ and $S_{2}$ denotes the sum over the tetrahedrons (Figure 6a). The symbol $\bar{N}$ is the mean value of the total number of tetrahedrons around a bond and is given by

(12) $$\begin{array}{c} \bar{N}=\left(1/N_{B}\right)\sum_{\mathrm{bond}\;i}\sum_{\mathrm{tet}(i)}1, \end{array}$$

where tet(i) is the total number of tetrahedrons around bond *i*, and $N_{B}$ is the total number of bonds. Due to this factor $1/\bar{N}$, the term $S_{1}$ is identical to $S_{1}\;=\;\sum_{ij}\ell_{ij}^{2}$, which is given by the sum of bond length squares $\ell_{ij}^{2}$. The reason why the summation convention $\left(1/\bar{N}\right)\sum_{\mathrm{tet}}$ is used instead of the simple form $\sum_{ij}$ for $S_{1}$ is that the discrete $S_{2}$ is naturally given by the sum over tetrahedrons as in the 2D case described above, and the same summation convention is also assumed for $S_{1}$. The symbol $\phi_{i}$ in $S_{3,i}$ is the internal angle of the triangle (Figure 6c), $\sum_{i}$ in $S_{3}$ is the sum over all internal angles *i*, and $\sigma_{1(i)}$ and $\sigma_{2(i)}$ in the definition of $S_{3,i}$ are σ at the bonds {1(i),2(i)} in which the internal angle is $\phi_{i}$ (Figure 6c). The symbol κ for the coefficient of $S_{3}$ is the same as the bending rigidity κ for $S_{3}$ of the 2D model in Equation (6); however, no confusion is expected. In the case of the 2D model, $S_{3}$ only resists pure bending. In contrast, $S_{3}$ of the 3D model in Equation (11) resists all deformations of the tetrahedron except the homologous deformation.

### 2.4. Simulation Technique

The standard Metropolis technique is used to update the variables $\vec{r}$ and σ [21,22]. For the update of ${\vec{r}}_{i}$ at vertex *i*, a new position ${\vec{r}}_{i}{\;}^{\prime }$ is generated by three uniform random numbers $\delta \vec{r}$ inside a sphere of fixed radius $r_{0}$ such that ${\vec{r}}_{i}{\;}^{\prime }\;=\;{\vec{r}}_{i}\;+\;r_{0}\delta \vec{r}$, where $r_{0}$ is a positive number. The new ${\vec{r}}_{i}{\;}^{\prime }$ is accepted with the probability Min[1,exp−δS], where δS is the change in the Hamiltonian due to the update of ${\vec{r}}_{i}$ such that δS=S(new)−S(old) under the constraints $U_{1}$ and $U_{2}$ on the bond length $\ell_{ij}$ and $\sigma_{i}$, respectively. The constraint $U_{2}$ is explicitly imposed on the update of σ, and this $U_{2}$ also implicitly imposes a constraint on the bond length $\ell_{ij}$. Indeed, the constraint $U_{2}$ on $\ell_{ij}$ in Equation (6) or Equation (11) for $\sigma_{ij}\;=\;1$ together with $U_{1}$ allows the bond length $\ell_{ij}$ to have only $\ell_{ij}\;=\;\ell_{ij}^{c}$ for the rigid bond model and $\ell_{ij}\;<\;\ell_{ij}^{c}$ for the empty bond model. The rate of acceptance for the update of $\vec{r}$ depends on the number $r_{0}$, which is fixed so that the acceptance rate is approximately in the range between 50% and 80%.

The update of $\sigma_{i}$ to the new $\sigma_{i}^{\prime }$ is performed independently of the old $\sigma_{i}$ with the probability Min[1,exp−δS], which is the same expression for the update of $\vec{r}$. In this update of $\sigma_{i}$, the change from $\sigma_{i}\;=\;0$ to $\sigma_{i}\;=\;1$, which is called crystallization, is allowed only when $\ell_{i}>\ell_{0}$. This crystallization process, from σ=0 to σ=1, causes a sudden decrease in $S_{i}\left(i\;=\;1,2\right)$ for both rigid and empty models. The energy $S_{3}$ also discontinuously decreases in the empty model, while it remains unchanged in the rigid bond model. Therefore, the crystallization process is determined only by $U_{i}\left(i\;\;=\;\;1,2\right)$ in both rigid and empty bond models. In contrast, the melting process, from σ=1 to σ=0, is not constrained by $U_{i}\left(i\;\;=\;\;1,2\right)$; therefore, this process is determined only by the energies $S_{i}\left(i\;\;=\;\;1,2,3\right)$ in both rigid and empty bond models. Indeed, in the rigid bond model, a sudden increase is expected in $S_{i}\left(i\;\;=\;\;1,2\right)$ for the melting process, and in the case of the empty bond model, a sudden increase is also expected in $S_{i}\left(i\;\;=\;\;1,2,3\right)$ for this process. Therefore, the melting process is determined only by the change in energy in both rigid and empty bond models according to the probability Min[1,exp−δS].

One more point on the update of σ that should be emphasized is that this update is performed only once every 100 Monte Carlo sweeps (MCSs), where one MCS is composed of *N* consecutive updates of $\vec{r}$. The reason why the variables $\vec{r}$ and σ are updated in such an asymmetrical manner is because a symmetrical or almost symmetrical update of σ causes a configuration with a nonuniform distribution of crystalline bonds, where “symmetrical” means that the total number of updates of $\vec{r}$ and σ in one MCS is the same. As a consequence of this nonuniform distribution of crystalline bonds, χ for the large strain region becomes lower than that for the intermediate strain region. This effect will be described in further detail in the presentation section.

### 2.5. Frame Tension as Tensile Stress

Detailed information on how to calculate the tensile stress is given in Ref. [20], and, here, we start with the formula. The formula for the stress in the 2D models is given by

(13) $$\begin{array}{c} \tau_{\mathrm{sim}}=\frac{2\langle S_{1}\rangle +4b\langle S_{2}\rangle -\left(3N^{\prime }-N_{\mathrm{bnd}}\right)}{2A}\frac{k_{B}T}{a^{3}}=\tau \frac{k_{B}T}{a^{3}}\;\left(\mathrm{Pa}\right), \end{array}$$

where $k_{B}$ and *T* are the Boltzmann constant and the temperature, respectively, A(=πDH) corresponds to the true area of the cylinder, and τ is the simulated frame tension. We should note that $3N^{\prime }-N_{\mathrm{bnd}}$ on the left-hand side should be replaced by $3N^{\prime }-2N_{\mathrm{bnd}}$ for the 3D model because the integration for the boundary vertices in *Z* of Equation (2) is the two-dimensional (one-dimensional) integration for the 2D (3D) model. The total number of degrees of freedom $3N^{\prime }$ is given by $3N^{\prime }\;=\;3N\;-\;N_{\mathrm{cr}}$ (rigid bond model) and $3N^{\prime }\;=\;3N\;-\;N_{\mathrm{cr}}/2$ (empty bond model). As mentioned in Section 2.2, the total number of degrees of freedom for the vertex move is slightly reduced by crystallization. For this reason, the number 3N in Equation (13) is replaced by $3N^{\prime }$.

**Figure 7:**
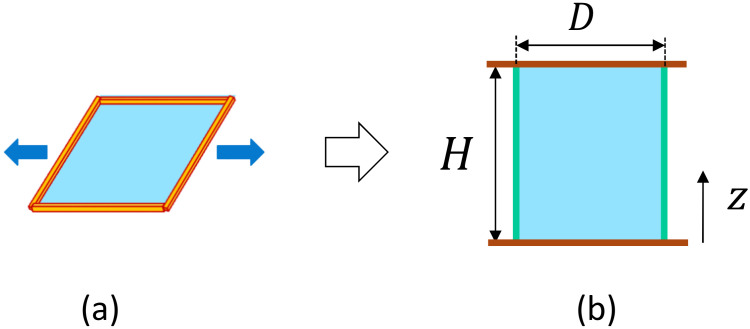
(**a**) Frame or projected area to be fixed to measure the surface tension τ, which can be called frame tension, in the case of a square surface, and (**b**) projected area A=πDH is fixed to calculate τ in the case of a cylindrical surface.

We briefly describe how to calculate the tensile stress $\tau_{\mathrm{sim}}$ in Equation (13) and how to change the unit from (N/m) to (Pa). The tensile stress is not directly calculated in our models because the materials represented by 2D or 3D lattices are very thin. Indeed, the 2D lattice is a cylindrical surface, and the 3D lattice is also regarded as a surface because its thickness is almost negligible compared to the diameter. In contrast, the frame tension τ is calculable as a response to a mechanical constraint, which is imposed by fixing the height *H* of the cylindrical surface (Figure 7a,b). For these reasons, τ is used to obtain $\tau_{\mathrm{sim}}$. The calculation formula of τ is actually obtained by a property of the partition function *Z* in Equation (2) under the scale change such as $\vec{r}\;\to \;\alpha \vec{r}\;\left(\alpha \;>\;0\right)$, which is a change of variables in the multiple-integration of *Z*. This property in *Z* is called scale invariance [23], and is expressed by Z(αr)=Z(r) for any α>0. From this, we have ${dZ\left(\alpha r\right)/d\alpha |}_{\alpha \to 1}\;=\;0$. This derivative of *Z* is directly calculated from the expression $Z\left(\alpha r\right)\;=\;\alpha^{3N-N_{\mathrm{bnd}}}\sum_{\sigma }\int \prod_{i=1}^{N-N_{\mathrm{bnd}}}d{\vec{r}}_{i}\prod_{i=1}^{N_{\mathrm{bnd}}}d{\vec{r}}_{i}\exp\left(-S\left(\alpha \vec{r},\sigma \right)\right)$, which is from the 2D model partition function in Equation (2), where the periodic boundary condition is assumed on the boundary vertices. Hence, the factor of α is calculated by $3\left(N\;-\;N_{\mathrm{bnd}}\right)\;+\;2N_{\mathrm{bnd}}\;=\;3N\;-\;N_{\mathrm{bnd}}$ from the fact that the boundary integration is two-dimensional. Note also that the total number of degrees of freedom 3N should be replaced by $3N^{\prime }$, as mentioned above. Except for the total number of degrees of freedom and difference in the symbols for energies, the calculation of ${dZ\left(\alpha r\right)/d\alpha |}_{\alpha \to 1}$ is exactly the same as those written in Ref. [20]. Thus, we obtain the surface tension $\tau \;=\;(2\langle S_{1}\rangle \;+\;4b\langle S_{2}\rangle \;-\;\left(3N^{\prime }\;-\;N_{\mathrm{bnd}}\right))/2A$. By multiplying $k_{B}T/a^{3}$ to this τ, we have the stress $\tau_{\mathrm{sim}}$ in Equation (13) in the unit of (Pa). The reason for the multiplication of $k_{B}T/a^{3}$ is as follows: First, we should note that the simulation unit is determined by fixing $k_{B}T\;=\;1$ (Nm) and a=1 (m), and, for this reason, all quantities with length units should be multiplied by the lattice spacing *a* in the physical unit. For this reason, by replacing the area *A* in the denominator of τ by $Aa^{2}$, we obtain $\tau /a^{2}$ with the unit of ($1/m^{2}$). Multiplying $k_{B}T$ to this $\tau /a^{2}$, we have $\tau k_{B}T/a^{2}$ with the unit of (N/m). This the physical unit of the frame tension τ. Finally, to obtain the quantity that has the unit of ($N/m^{2}$), we have to multiply 1/a to this quantity once again, and this leads to the expression of $\tau_{\mathrm{sim}}$ in Equation (13).

By assuming room temperature for *T*, we have

(14) $$\tau_{\mathrm{sim}}=\left(4\times 10^{-21}\right)\frac{\tau }{a^{3}}\;\left(\mathrm{Pa}\right).$$

The symbol a(m) is the lattice spacing or the bond length, which corresponds to the coarse-grained distance between polymer segments, and, hence, it can be fixed to an arbitrary number larger than the van der Waals distance (∼$10^{-10}\;\left(m\right)$). This implies that the magnitude of the calculated τ can be controlled arbitrarily such that $\tau_{\mathrm{sim}}$ is comparable to experimental data $\tau_{\exp}$ by using this parameter $a(>\;10^{-10})$.

The diameter *D*, which is equal to the initial height $H_{0}$, is fixed such that the height strain

(15) $$\varepsilon =H/H_{0}-1$$

is zero for $H\;=\;H_{0}$. For this purpose, MC simulations should be performed to obtain the correct value of $H_{0}$, which depends on the other parameters, such as *b* and κ, before the start of the production simulations.

Once $H_{0}$ is obtained, the next task that should be done before the production simulations is to fix the constant $\ell_{0}$ in $U_{1}$ of Equation (7). Using the value of $H_{0}$, we fix $\ell_{0}$ to

(16) $$\begin{array}{c} \ell_{0}\;=\;H_{0}/10 \end{array}$$

for both the 2D lattice of size N= 10,230 and the 3D lattice of size N=9760. This value of $\ell_{0}$ is crucial to the shape of the crystallization ratio χ vs. strain, which influences the final result of the stress–strain diagram. However, the results are almost independent of a small variation of this value of $\ell_{0}$. The factor 1/10 in $\ell_{0}\;=\;H_{0}/10$ is expected to be dependent on the size *N* in general because $H_{0}$ depends on $L_{1}$ as in Equation (1), and this $L_{1}$ determines *N* such that $L_{1}\;=\;15$ for the 2D lattice of size N= 10,230 and $L_{1}\;=\;8$ for the 3D lattice of size N=9760. We should note that the value of $\ell_{0}$ or $H_{0}$ depends on the experimental data of stress–strain curves to be fitted and on whether the model is 2D or 3D. In this paper, we assume Equation (16) for $\ell_{0}$ because the experimental data of stress–strain curves are well reproduced, as we see in the following section.

## 3. Monte Carlo Results

### 3.1. Snapshots

**Figure 8:**
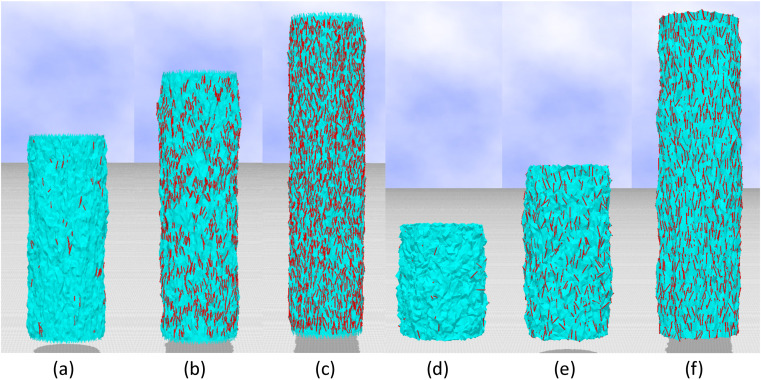
Snapshots of the (**a**–**c**) 2D empty model and (**d**–**f**) 3D model, where small red lines denote the crystalline bonds. The crystallization ratio is (**a**) χ≃0.0035, (**b**) χ≃0.060, (**c**) χ≃0.12, (**d**) χ≃0.0016, (**e**) χ≃0.026, and (**f**) χ≃0.083.

Snapshots for the 2D and 3D empty models are shown in Figure 8a–f, respectively. Small red lines denote the crystalline bonds corresponding to σ=1. The crystallization ratio χ for the 2D empty model in Figure 8a–c is χ≃0.0035, χ≃0.060, and χ≃0.12, respectively, and χ for the 3D model in Figure 8d–f is χ≃0.0016, χ≃0.026, and χ≃0.083, respectively. The value of χ≃0.12 in Figure 8c for the 2D model is close to $\chi_{\max}\;\;≃\;\;0.15$, and χ≃0.083 in Figure 8f for the 3D model is also close to $\chi_{\max}\;\;≃\;\;0.09$. We should note that the crystalline bonds are uniformly distributed in both the outer and inner surfaces in the case of the 3D model.

### 3.2. Results of 2D Models

Pradhan et al. [24] synthesized elastomer nanocomposites with nanoparticle dispersion and studied their mechanical properties. The strain of the carboxylated nitrile rubber (XNBR) with layered double hydroxide (LDH) is reported to be very large (∼1000% or more), and the stress–strain data are influenced by SIC; hence, XNBR/LDH is similar to natural rubber. For this reason, we compare our simulation results with the experimental data of these materials denoted by XL5 and XL10 in Ref. [24]. The numbers 5 and 10 denote the LDH content with the units of phr, parts per hundred rubber.

The reason why these experimental data, reported in [24], are compared with the simulation data is that these reported data are considered to be of an equilibrium state. In contrast, experimental data of natural rubber such as those plotted in Figure 1c are not of equilibrium state, although the data obtained in the unloading process are almost close to those of the equilibrium state.

As described in the introduction, SIC causes a plateau in the intermediate strain region of stress–strain curves, and, moreover, an upturn of the curve or hardening is observed in the large strain region where the plateau terminates. This upturn is also due to the limitation of extensibility of the polymer chains [24]. For this reason, the authors in Ref. [24] used a modified Mooney–Rivlin equation [25,26] to see whether SIC truly influences the stress–strain curves, and found that XL5 and XL10 are influenced by SIC. Here, we should note that, as mentioned in the introduction, we focus on the stress relaxation in this paper. For this reason, the upturn in the large strain region is simply implemented in the models by the term $S_{2}$, which is quadratic with respect to the bond length $\ell_{ij}$, and, therefore, it is almost independent of crystalline bonds.

**Figure 9:**
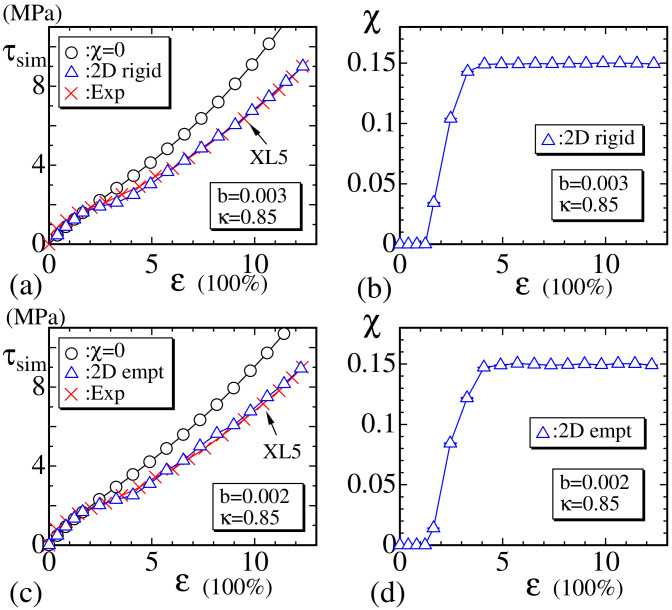
(**a**) The stress $\tau_{\mathrm{sim}}$ vs. strain ε obtained by the 2D rigid model for the experimental data XL5 ($\begin{array}{c} \times \end{array}$) and (**b**) the corresponding χ vs. ε; (**c**) $\tau_{\mathrm{sim}}$ vs. ε obtained by the 2D empty model and (**d**) the corresponding χ vs. ε. In both (a) and (c), the symbol (◯) denotes the data without crystallization obtained by assuming the same values of parameters *b* and κ assumed for the data ($\begin{array}{c} △ \end{array}$).

In Figure 9a, the experimental data ($\begin{array}{c} \times \end{array}$:Exp) of XL5 and the simulation results (◯ and $\begin{array}{c} △ \end{array}$) of the 2D rigid bond model are plotted. The parameters *b* and κ are fixed to b=0.003 and κ=0.85, respectively. The coefficient *b* of $S_{2}$, which is the quadratic part of tensile energy, is fixed to a nonzero value, though it is very small compared to the coefficient 1 of $S_{1}$. This small but nonzero *b* plays a role in the large strain region to increase $\tau_{\mathrm{sim}}$ in both models, as mentioned above. The assumed value of $\ell_{0}$ in Equation (16) for 2D models is given by

(17) $$\ell_{0}=2.43a\;\left(2D\;\mathrm{rigid},\;\mathrm{XL}5\right),\;\ell_{0}=2.45a\;\left(2D\;\mathrm{empty},\;\mathrm{XL}5\right).$$

The simulation data (◯) correspond to χ=0, which implies that no crystalline bond is included. We find that no plateau region is included in the data of χ=0. In contrast, the curve of data ($\begin{array}{c} △ \end{array}$) is clearly different from (◯) and has a small plateau at the region 2<ε<4, where χ plotted in Figure 9b increases from χ=0 to $\chi_{\max}\left(≃\;0.15\right)$. This result implies that τ decreases with increasing χ, and, hence, this corresponds to the stress relaxation induced by SIC. The reason why τ ($\begin{array}{c} △ \end{array}$) becomes lower than τ (◯) is very simple to understand because the crystalline bond is defined to have no tensile energy, and, therefore, $\langle S_{1}\rangle$, $\langle S_{2}\rangle$, and $3N^{\prime }$ in $\tau_{\mathrm{sim}}$ of Equation (13) decrease with increasing χ. The results of the 2D empty bond model are plotted in Figure 9c,d, which are almost the same as those of the 2D rigid bond model shown in Figure 9a,b. The parameters *b* and κ in the 2D empty model are fixed to b=0.002 and κ=0.85, respectively, which are almost the same as those fixed in the 2D rigid model.

Thus, the stress relaxation is understood to be a result of the fact that the total tensile energy accumulated in the amorphous part is simply decreased by crystallization in real materials. Indeed, the total number of segments of the amorphous part that shares the tensile energy is decreased by SIC in the models. Moreover, in real materials, at the small strain region where the crystallization starts to increase, almost no accumulation of the tensile energy is expected in the crystalline part. This property in real materials is shared by the models in this paper. However, the tensile energy shared by the crystalline part cannot be neglected for the large strain region, where the amorphous part is almost maximally extended and has no room for the accumulation of tensile energy. This property is not shared by the models in this paper, and, for this reason, the sudden upturn by SIC at the large strain region is not reproduced.

We consider that a part of the difference between the curves (◯) and ($\begin{array}{c} △ \end{array}$) corresponds to the entropy change. The temperature in the recovery process is also expected to be lower than that in the extension process in real experiments, and this temperature difference is due to the decrease in entropy from the thermodynamics viewpoint. The decrease in entropy from the microscopic viewpoint is naturally expected from our models because the crystalline bond of σ=1 effectively decreases the total number of degrees of freedom for the variables ${\vec{r}}_{i}$, as mentioned in Section 2.2.

**Figure 10:**
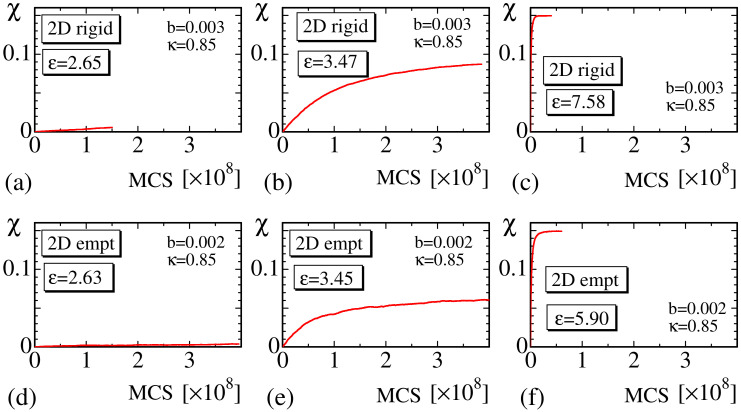
The crystallization ratio χ vs. Monte Carlo sweep (MCS) of the 2D rigid model obtained at (**a**) ε≃2.65, (**b**) ε≃3.47, and (**c**) ε≃7.58, and the ratio χ vs. MCS of the 2D empty model at (**d**) ε≃2.63, (**e**) ε≃3.45, and (**f**) ε≃5.90.

In Figure 10a–f, χ vs. MCS are plotted, and the convergence of MC simulations for 2D rigid and empty bond models are shown. We find that in both models, the convergence at the transition region (Figure 10b,e), where χ has an intermediate value between 0 and $\chi_{\max}$, is relatively slower than those at small (Figure 10a,d) and large strain regions (Figure 10c,f). The slow speed of convergence at the intermediate strain region is reasonable because crystallization is understood to be a first-order transition. One more point to note is that χ has an intermediate value between χ=0 and $\chi_{\max}$ in Figure 10b,e. This observation indicates that χ smoothly changes with respect to strain ε even though SIC is a first-order transition. We will not go into detail on this first-order transition because our focus in this paper is not on the first-order nature of the crystallization.

Finally, in this subsection, we should comment on the reason for why the MC update of σ is performed only once every 100 MCSs. As mentioned in Section 2.4, the crystallization ratio χ at the large strain region becomes slightly smaller than $\chi_{\max}$ if the MC update is performed more frequently. This result comes from the fact that the convergence speed of the variable $\vec{r}$ is very slow compared with that of σ. In other words, if σ is updated more frequently before the lattice configuration is equilibrated, then the crystalline bonds of σ=1 are expected to be distributed non-uniformly, and χ will be smaller than $\chi_{\max}$ at the large strain region.

### 3.3. Results of the 3D Model

**Figure 11:**
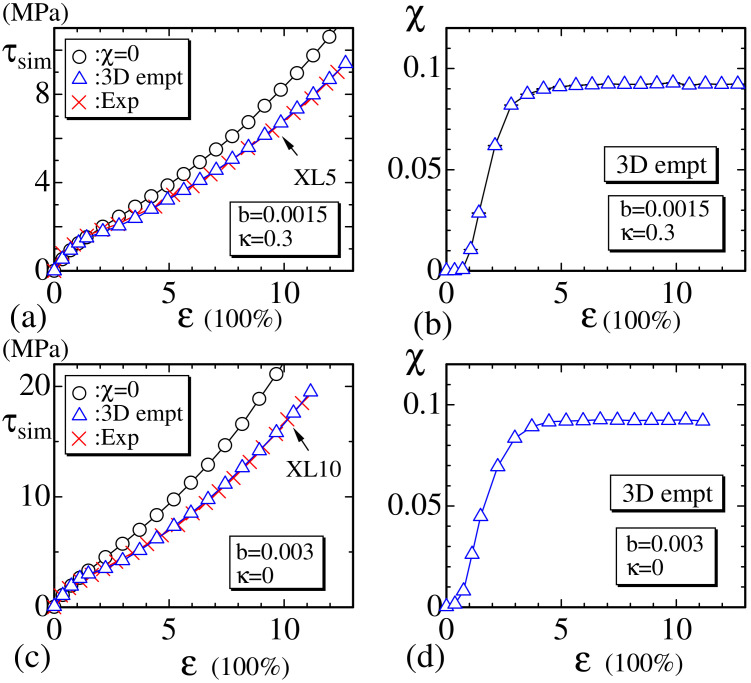
(**a**) The stress $\tau_{\mathrm{sim}}$ vs. strain ε obtained by the 3D empty model for the experimental data XL5 ($\begin{array}{c} \times \end{array}$) and (**b**) the corresponding χ vs. ε; (**c**) $\tau_{\mathrm{sim}}$ vs. ε obtained by the 3D empty model for the experimental data XL10 ($\begin{array}{c} \times \end{array}$) and (**d**) the corresponding χ vs. ε. In both (a) and (c), the symbol (◯) denotes the data without crystallization obtained by assuming the same values of parameters *b* and κ.

Now, we show the simulation results of the 3D empty bond model. The main difference between 3D and 2D models is that the Hamiltonian depends on the dimension D, and this difference in dimension is a huge difference in general from the modeling view point, and, therefore, it is interesting to see whether or how the results change depending on the dimension D. The reason for why only the empty bond model is simulated in the 3D case is because no specific difference is found between the two models, rigid and empty, in the 2D case. The obtained results $\tau_{\mathrm{sim}}$ and χ are plotted in Figure 11a–d together with the reported experimental data. The symbols ($\begin{array}{c} \times \end{array}$:Exp) in Figure 11a,c correspond to the experimental data denoted by XL5 and XL10, respectively [24]. The parameters (b,κ) are fixed to (b,κ)=(0.0015,0.3) and (b,κ)=(0.003,0) for the experimental data XL5 and XL10, respectively. The value of $\ell_{0}$ in Equation (16) for the 3D model is given by

(18) $$\ell_{0}=2.69a\;\left(3D\;\mathrm{empty},\;\mathrm{XL}5\right),\;\ell_{0}=2.84a\;\left(3D\;\mathrm{empty},\;\mathrm{XL}10\right).$$

We find that the simulation results by the 3D empty model are in good agreement with the experimental data, just as in the case of the 2D models.

**Figure 12:**
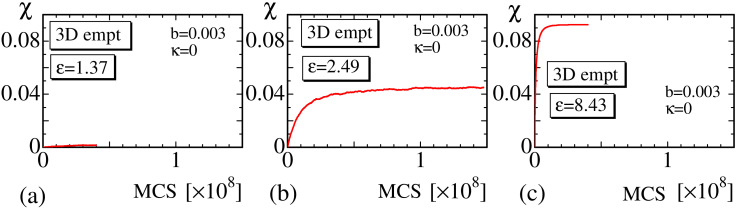
The crystallization ratio χ vs. MCS of the 3D empty model obtained at (**a**) ε≃1.37, (**b**) ε≃2.49, and (**c**) ε≃8.43.

We plot χ vs. MCS obtained at the small, intermediate, and large strain regions in Figure 12a–c to see the convergence of the MC simulations. It is clear that the convergence speed in the intermediate strain region is also slower than that in the small and large strain regions, as in the 2D models. Note also that the convergence speed of the 3D model is relatively faster than that of the 2D models. We consider that this result comes from the fact that the surface or lattice fluctuation of the 3D models is relatively smaller than that of the 2D model, or, in other words, the phase space volume for the MC update of the polymer position $r_{i}$ is expected to be relatively smaller in the 3D model than in the 2D models. Indeed, the 2D lattice is composed of triangles, and, hence, the vertex position easily moves even when κ is fixed to κ=0.85. In contrast, the 3D lattice is composed of tetrahedrons, and, hence, the vertices hardly move compared to those on the 2D lattice.

Next, we calculate the assumed lattice spacing *a* to calculate τ, as plotted in Figure 9 and Figure 10. The results are shown in Table 1. The lattice spacing is considered to be the unit of distance between two neighboring vertices, which are understood to be coarse-grained lengths of polymer segments (see Equations (17) and (18)). Therefore, the value of *a* should be at least larger than the van der Waals distance (≃$10^{-10}\;\left[m\right]$). From Table 1, we find that all assumed *a* values are larger than $10^{-10}\;\left[m\right]$, and, therefore, lattice modeling is meaningful as a coarse-grained technique for polymer networks.

**Figure 13:**
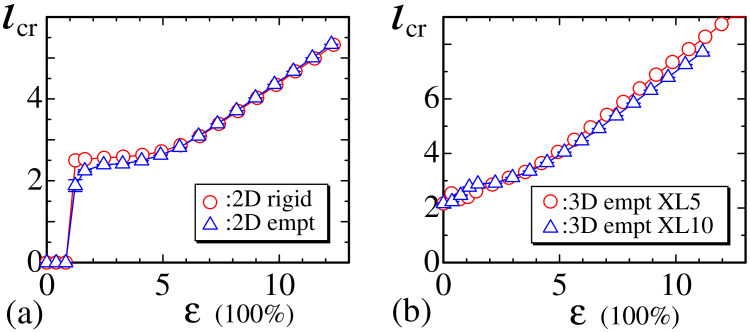
The mean length $\ell_{\mathrm{cr}}$ of crystalline bonds corresponding to (**a**) 2D rigid and empty models and (**b**) the 3D empty model for the experimental data XL5 and XL10.

Finally, in this subsection, we plot the mean length $\ell_{\mathrm{cr}}$ for crystalline bonds in Figure 13a,b. This $\ell_{\mathrm{cr}}$ is not always identical to $\ell_{i}^{c}$ in Equation (7) because of the constraint $\ell_{\mathrm{cr}}<\ell_{i}^{c}$ for the crystalline bond length in the empty bond model. However, as described in Section 2.2, $\ell_{i}^{c}$ is dynamically changeable, and, hence, $\ell_{\mathrm{cr}}$ is also dynamically changeable and depends on the strain ε. The sudden jump of $\ell_{i}^{c}$ in Figure 13a for the 2D models implies that there are no crystalline bonds in the small strain region close to ε≃0, and the nonzero $\ell_{i}^{c}$ in Figure 13b implies that there is more than one crystalline bond even in the small strain region in the case of the 3D model. We find that $\ell_{i}^{c}$ increases with increasing ε, and this behavior is consistent with that observed in real materials [3].

### 3.4. Dependence on Stiffness κ

**Figure 14:**
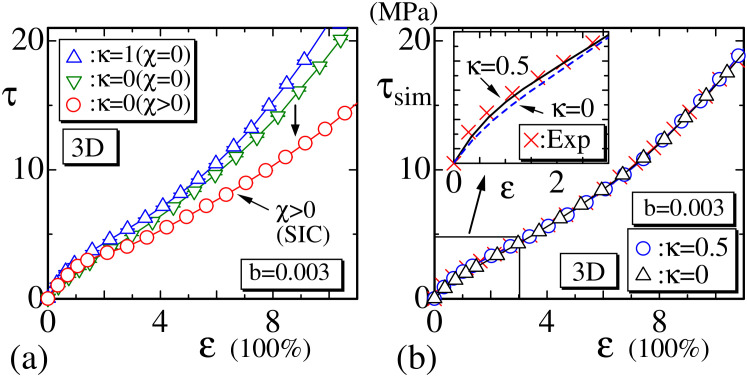
(**a**) The stress τ vs. ε obtained by the 3D model with the crystalline state (◯) and without the crystalline state (▵, ▿), and (**b**) the stress $\tau_{\mathrm{sim}}$ vs. ε obtained without the crystalline state. The stress τ in (**a**) has the simulation unit, which corresponds to $k_{B}T\;=\;1$ and is not modified by the factor $k_{B}T/a^{3}$ in Equation (13), while the stress $\tau_{\mathrm{sim}}$ in (b) has the real physical unit (MPa) and is modified by the factor $k_{B}T/a^{3}$. Experimental data denoted by Exp (×) in (**b**) are XL5, the same as in Figure 11c.

In the preceding subsection, two different experimental data, XL5 and XL10, are simulated and plotted in Figure 11a,c, where the stiffness κ=0.3 and κ=0 are assumed, respectively. However, the dependence of the results on κ is not so clear. Therefore, in this subsection, we should discuss the effect of stiffness κ on the stress–strain curve and clarify that the effect of κ is different from the effect of SIC. First, we plot in Figure 14a the results of 3D model with the crystalline state (◯) (⇔χ>0) and those without the crystalline state (▵, ▿) (⇔χ=0), where κ is fixed to κ=1 and κ=0, respectively. In Figure 14a, the values of τ are given by Equation (13) without the factor $k_{B}T/a^{3}$, and these are raw simulation data expressed by the simulation unit. It is clear from Figure 14a that the data (◯) decrease in the intermediate strain region and are smaller than the other two data (▵, ▿) of χ=0, except in the smaller strain region. This decrement of τ (◯) is understood to be due to the crystalline states.

To see the effect of κ more clearly, the stress $\tau_{\mathrm{sim}}$, which includes the factor $k_{B}T/a^{3}$ in Equation (13), is plotted in Figure 14b with the experimental data (×). Inside Figure 14b, the data in the small strain region are drawn by solid and dashed lines. We find from these solid and dashed lines that the increment of κ increases the stress only at the region ε≤2.5. On the other hand, SIC has no effect on the stress in the small strain region in general, because SIC is effective only at the large strain region, such as ε≥2.5, for example. Thus, we find from these numerical data that the stress relaxation by SIC is independent of the effect of κ. In other words, the stress relaxation by SIC, observed in experimental data, is unable to be understood without the crystalline states, such as the rigid or empty bond introduced in Section 2.2, at least in the framework of standard Gaussian chain models.

We also find that the results of the 2D models without crystalline states are completely inconsistent with the experimental data even when κ is increased to be sufficiently large. This means that, in the case of 2D models, experimental data can only be reproduced with the crystalline states, which are introduced by rigid bonds or empty bonds in Equations (4) and (5).

## 4. Summary and Conclusions

We have studied the equilibrium property of stress relaxation observed in rubbers undergoing strain-induced crystallization (SIC) by Monte Carlo (MC) simulations for simple models constructed by extending the Gaussian chain model. Two types of new models, rigid and empty, are introduced and studied. In these models, a new variable σ is introduced, and it is defined on the bond to distinguish the amorphous and crystalline states. The crystalline state is simply defined to have no tensile energy, where the tensile energy is given by a spring potential or the so-called Gaussian bond potential and is defined only on the amorphous state.

We find that the obtained MC results for the stress–strain curves are in good agreement with the existing experimental data of composite materials, of which the mechanical property was reported to be close to that of natural rubber, and the corresponding stress–strain curves are influenced by SIC [24]. Here, we have to emphasize that these experimental data are not time-dependent ones, which can be considered the equilibrium ones, and, hence, can be close to unloading stress–strain curves. In the simulation data, stress relaxation is clearly observed in the intermediate strain region, where the crystallization ratio χ starts to increase. The fact that no tensile energy is accumulated in the crystalline bonds in our models indicates that the stress relaxation is simply due to the decrease in the tensile energy of the amorphous state, where this decrease in energy is in the sense that the total energy is decreased due to the change in state from amorphous to crystalline. No contribution to the tensile energy is expected from the crystalline state, at least in the intermediate strain region.

In this paper, we focus only on stress relaxation, and the upturn of the stress–strain curve or hardening at the large strain region is implemented simply by including a quadratic term in the tensile energy, which is not directly connected to the crystalline state. This upturn of the stress–strain curve is another interesting phenomenon associated with SIC, and, hence, it remains to be studied more intuitively from a microscopic viewpoint.

## Figures and Tables

**Table 1:** The lattice spacing *a* used for $\tau_{\mathrm{sim}}$ (in Equation (14)) plotted in Figure 9 and Figure 11.

Figures	Model	*a*[m] for Data (◯)	*a*[m] for Data (△)
Figure 9a	2D rigid	$1.305\times 10^{-9}$	$1.307\times 10^{-9}$
Figure 9c	2D empty	$1.329\times 10^{-9}$	$1.365\times 10^{-9}$
Figure 11a	3D empty	$1.265\times 10^{-9}$	$1.269\times 10^{-9}$
Figure 11c	3D empty	$1.654\times 10^{-9}$	$1.629\times 10^{-9}$

## Abbreviations

The following abbreviations are used in this manuscript:

2D	two-dimensional
3D	three-dimensional
MC	Monte Carlo
MCS	Monte Carlo Sweep
SIC	Strain-Induced Crystallization
